# Further characterization of “subject’s own name (SON) negativity,” an ERP component reflecting early preattentive detection of SON

**DOI:** 10.1186/s13104-015-1150-8

**Published:** 2015-05-12

**Authors:** Toshihiko Tateuchi, Kosuke Itoh, Tsutomu Nakada

**Affiliations:** Forensic Science Laboratory, Chiba Prefectural Police HQ, Chiba, Japan; Center for Integrated Human Brain Science, Brain Research Institute, University of Niigata, 1-757 Asahimachi, Niigata, 951-8585 Japan

**Keywords:** Attention, Orienting response, Familiarity, Event-related potentials, The self, Self reference

## Abstract

**Background:**

Subject’s own name (SON) is detected automatically and unconsciously in the brain. SON negativity, an early wave in the mismatch negativity latency range, has been proposed as a potential event-related potential (ERP) index of the automatic preattentive detection of SON. SON negativity is probably not a general measure of familiarity, as it is not elicited by the subject’s parent’s name. We further investigated the specificity of this response by testing whether it is elicited by a name to which subjects were strongly but only temporarily familiarized.

**Findings:**

Subjects performed a task to detect an arbitrary unfamiliar name for forty minutes. Then, that name was presented randomly and equiprobably with nine novel unfamiliar names while they played a video game and tried to ignore the sounds. SON negativity was not elicited, even when subjects spontaneously noticed hearing the familiarized name.

**Conclusions:**

The finding supports the notion that SON negativity represents a specific ERP measure of the early preattentive detection of SON, rather than a general measure of familiarity.

## Findings

### Introduction

Subject’s own name (SON) is a unique auditory stimulus that is processed distinctively from other sounds in the human brain. SON, for example, serves as an exceptionally effective stimulus for triggering an orienting response, i.e., an involuntary capture of attention by an external input [1,2]. While sounds in general need to stand out clearly from the background in one or more physical features (such as loudness) to elicit an orienting response, a quiet whispering of SON by a third irrelevant person is often sufficient to cause an involuntary attention capture. Event-related potential (ERP) recordings have shown that SON is distinguished from other names not only during wakefulness [3-8] but also during sleep [9,10]. Moreover, SON elicits distinctive neural activities in brain-damaged patients with altered states of consciousness [11-16]. These observations are consistent with the hypothesis that the brain has some specialized neural mechanisms to distinguish SON from other sounds “unconsciously” and “automatically” in an early preattentive stage of auditory processing [8].

A previous experiment identified an ERP component labeled “SON negativity” as a potential index of the automatic preattentive detection of SON [8]. In that study, various name stimuli were presented to the subjects while they played a video game and ignored the sounds. SON consistently elicited an early frontal negativity (SON negativity, 170–270 ms) in the latency range of mismatch negativity (MMN) [17], whether or not the presentation frequency of SON was high (~50%) or low (10%). Following SON negativity in latency, a P3a-like frontal positivity signifying orienting response was elicited by SON only when it was the rare stimulus, and its amplitude decreased with the repeated presentation of SON. In other words, an early preattentive processing of speech sounds distinguished SON from other names, and this later culminated in an orienting response only when SON was contextually informational.

These results suggested the following two-stage neural processes underlying the orienting response to SON [8]. First, SON is automatically distinguished from other names in an early stage of cortical processing, without reaching awareness (i.e., preattentively) in a manner independent of the short-term probability (or short-term information value) of SON. Second, this is followed by an orienting response to cause a shift of attention, when and only when the preattentively detected SON is evaluated as being contextually informative, such as when SON is the rare stimulus in an oddball paradigm. In the ERP, SON negativity reflects the early automatic preattentive detection of SON, while P3a indexes the actual occurrence of an orienting response.

In the aforementioned experiment, SON negativity was not elicited by the subject’s parent’s name, which is a name that has been made personally meaningful to the subject in his or her long-term memory [8]. In the present study, we further investigated the specificity of SON negativity by testing whether it is elicited by a name that is made personally meaningful to the subject in his or her short-term memory. A negative finding would corroborate the hypothesis that the brain has acquired some mechanisms to distinguish SON from other names preattentively, and that SON negativity represents a specific index of this auditory function rather than a general measure of familiarity. Subjects first performed a button-press task to detect an arbitrarily chosen unfamiliar name for forty minutes. Then, the familiarized target name was presented randomly and equiprobably with nine other novel unfamiliar names (10% each), while they played a video game with an instruction to ignore the sounds. Post-experiment surprise interviews asked whether they spontaneously noticed hearing the familiarized target name, and the ERPs to the name stimuli were analyzed with special attention to the latency range of SON negativity.

## Methods

### Subjects

Seventeen neurologically and audiologically normal volunteers (22.1 ± 1.9 s.d. years old, eight men and nine women) participated in the study after giving their informed consent. Studies were performed according to the human research guidelines of the Internal Review Board of University of Niigata. All were right-handed as confirmed by the Edinburgh handedness inventory [18].

### Materials and procedure

Stimuli were common given names of the same sex as the subject, which were preselected for each subject to exclude familiar person’s names (e.g., family members, relatives, close friends, etc.). All name stimuli were spoken by a same single male, digitally recorded, and edited in amplitude to have matched subjective loudness as judged by two authors (TT and KI). Durations of the stimuli were 2–5 syllables, or approximately 250–600 ms long. The stimuli were presented binaurally at about 65 dB SPL, with a randomly varied stimulus onset asynchrony (SOA) of 1200–1400 ms, using STIM software and hardware (Neuroscan).

Frist, subjects performed a simple name detection task, in which they pressed a button, as fast as possible, to an arbitrarily chosen unfamiliar name (referred to as TARGET) that was randomly and equiprobably (10%) presented with nine other unfamiliar names. One continuous block of the task comprised 350 trials and lasted about 8 minutes. A total of five blocks were administered to each subject, with intervening short breaks. The TARGET and other name stimuli were identical throughout the five blocks in each subject, but they varied across subjects to minimize possible confound related to word length, sound spectra, or other physical properties of the stimuli.

After completing the detection task, ERPs to the name stimuli were recorded while the subjects played a cognitively demanding video game (Tetris) on a portable game machine (Gameboy, Nintendo, Kyoto, Japan) with an instruction to ignore the auditory stimuli. Ten name stimuli were presented in a randomized order. One of the stimuli was the familiarized TARGET that was used in the detection task, and the other nine were novel unfamiliar names that were not used in the detection task (referred to as NON-TARGET). The TARGET and NON-TARGET stimuli were presented equiprobably (10% each) in a continuous block of 350 trials, and two blocks were administered for each subject. At the beginning of each block, ten unfamiliar names, which were all different from the TARGET and NON-TARGETS, were presented, and the data obtained during this period were discarded to reduce possible effects of subject’s attention being captured by the initiation of the stimulus train.

After finishing recording, subjects were administered a (surprise) free recall test in which they wrote down on a blank sheet of paper the names that they noticed hearing during playing game.

### EEG recording and analysis

Electroencephalograms (EEG) were recorded from 21 Ag electrodes (Fp1, Fp2, F3, F4, F7,F8, C3, C4, P3, P4, T3, T4, T5, T6, O1, O2, Fpz, Fz, Cz, Pz, Oz) positioned on the scalp according to the International 10–20 System. Horizontal (HEOG) and vertical (VEOG) electro-oculograms (EOG) were also recorded from the left eye. All EEG and EOG signals were referenced to linked ears, amplified (×500), bandpass filtered (0.05-100 Hz), and sampled at 1000 Hz using SynAmps (Neuroscan). Electrode impedance was kept below 5 kΩ.

To obtain ERPs, the EEG data were segmented time-locked to stimulus onset (−200 to 1200 ms), baseline corrected using the pre-stimulus period average, low-pass filtered at 30 Hz (48 dB/oct), artifact rejected at ±100 μV, and averaged for each stimulus and condition. Effects of stimulus on the ERP amplitudes were analyzed by a two-way repeated-measures analysis of variance (ANOVA) with factors Stimulus (TARGET/NON-TARGET) and Electrode (21 EEG channels). The *p* values were adjusted by Huynh-Feldt correction whenever necessary, and *ε* is reported in such cases. Effect sizes for the ANOVAs are shown in terms of partial eta squared (*η*_p_^2^).

## Results

### Behavior

In the free recall test administered after the ERP recording, nine out of seventeen subjects reported hearing the TARGET during playing game, despite the experimenter’s instruction to ignore the sounds. Based on this result, all data were analyzed separately for the two groups: NOTICED (*n* = 9), who spontaneously noticed hearing the familiarized name, and UNNOTICED (*n* = 8), who did not. Because the purpose of the name detection task was to familiarize a previously unfamiliar name to a level that it is clearly distinguished from other unfamiliar names, the NOTICED group represented the main focus of our ERP analyses. Nonetheless, results for the UNNOTICED group are also presented for comparison.

In the name detection task conducted prior to ERP recording, the average (± standard deviations, s.d.) hit rate for TARGET was 99.1 ± 1.7% in the NOTICED group and 96.2 ± 6.2% in the UNNOTICED group; the difference was not significant, *t*(15) = 1.4, *p* = 0.20. The mean false alarm rates were 0.2 ± 0.2% and 0.3 ± 0.1% for the NOTICED and UNNOTICED groups, respectively. The mean reaction time for the correct detection of TARGET was 554 ± 91 ms in the NOTICED group and 546 ± 115 ms in the UNNOTICED group, which were not significantly different from each other, *t*(15) = 0.1, *p* = 0.88.

### ERP

**Figure 1:**
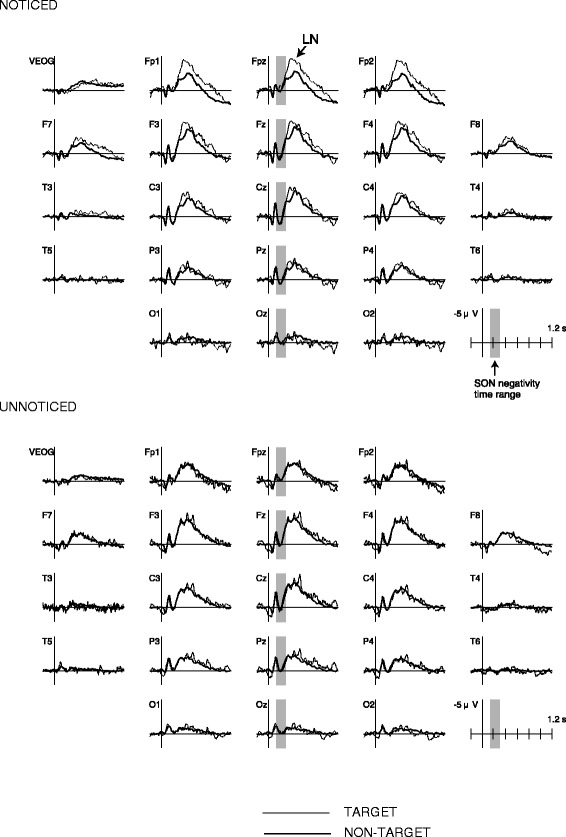
ERP to name stimuli. Traces show the ERPs to a temporarily familiarized TARGET name (thin lines) and unfamiliar NON-TARGET names (thick lines) in subjects who spontaneously noticed hearing the TARGET during playing video game (NOTICED), and in those who did not (UNNOTICED). The neural responses to TARGET and NON-TARGET were not distinguishable in the latency range of SON negativity (about 150–300 ms, shaded region) in either subject group. In the NOTICED group, TARGET elicited a late negativity (LN, 300- ms) over frontal and frontal polar regions.

Figure 1 shows the ERPs for the TARGET and NON-TARGET names, separately for the NOTICED and UNNOTICED groups. In either group, no hint or evidence for an elicitation of SON negativity was found in its reported latency range of about 150–300 ms. After this latency, however, TARGET elicited a late negativity (LN) in the NOTICED group (Figure 1). LN was maximal in amplitude at the frontal polar region, peaked around 390 ms, and lasted about 1 s in duration. An ANOVA performed on the averaged amplitude in 360–420 ms time range in the NOTICED group revealed a significant Stimulus × Electrode interaction, *F*(20, 160) = 3.5, *p* < 0.05, *ε* = 0.12, *η*_p_^2^ = 0.30. In a follow-up ANOVA at the Fpz electrode, where the LN amplitude was maximal, the simple main effect of Stimulus was significant, *F*(1, 8) = 6.5, *p* < 0.05, *η*_p_^2^ = 0.45. LN was not apparent in the UNNOTICED group (Figure 1).

## Discussion

The underlying hypothesis of this study was that SON is detected preattentively in auditory processing by some specialized neural mechanisms, and that SON negativity serves as a specific ERP index of this auditory function. A previous experiment showed that SON negativity is not elicited by the subject’s parent’s name, which is one of the most suitable stimuli other than SON for testing long-term familiarity effects on the neural processing of names [8]. On the other hand, the present study investigated the possible short term familiarity effects on the neural processing of names, by artificially making a previously unfamiliar name temporarily familiar to the subject. Our name detection task was at least partly successful for this purpose, in that the TARGET was spontaneously detected by some subjects while they played a cognitively demanding video game. Nonetheless, the familiarized TARGET name did not elicit a SON negativity in these subjects. Results thus corroborated the notion that SON negativity is a specific index of early automatic SON detection, rather than a general measure of familiarity effects on the neural processing of names. Further studies are necessary to clarify what attributes of the SON stimulus, e.g., extensive exposure from infancy, emotional valence, personal relevance, self-awareness, etc., are important in eliciting this early brain response (e.g., [19-22]).

The familiarized TARGET name elicited an LN in the NOTICED group. In the UNNOTICED group that did not hear the TARGET, by contrast, the ERPs to TARGET and NON-TARGET were virtually indistinguishable in the LN (as well as other) latency range(s). In other words, LN reflected detection of familiarized TARGET by neural activities that occurred more slowly than the preattentive detection of SON. Intriguingly, the LN recorded in the present experiment was very similar in latency and scalp distribution to the LN elicited by subject’s parent’s name [8]. There appears to be shared neural processes for the detection of familiar names stored in long-term memory and those stored in short-term memory. LN probably serves as a general measure of familiarity effects on the cerebral cortical processing of names, which is in sharp contrast to SON negativity. Another more general interpretation of LN is that it is elicited nonspecifically by any noticeable auditory stimuli. Further studies are warranted that clarify the functional significance of LN.

Does the SON negativity represent a distinct ERP component, rather than a modulation of some other ERP components in the same time range? The SON negativity overlapped the N1-P2-N2 responses [8], and it could have been possible to interpret this negativity as a diminution in amplitude of P2, for example. This view, however, had a couple of difficulties. First, the negative shift of potential (i.e., the SON negativity) occurred not only in the restricted time range of P2, but more broadly into the latency ranges of N1 and N2 [8]. To explain this phenomenon, it was simpler to hypothesize an overlap of a single negativity than to posit that three different phenomena occurred simultaneously: N1 increase, P2 decrease, and N2 increase. Second, SON negativity and P2 had different scalp topographies. While the SON negativity was maximal at Fz and had a frontocentral distribution [8], P2 is typically more clearly central and is maximal at the vertex for a range of different types of stimuli, including verbal sounds [23-26]. Thus, the generators of P2 and SON negativity were probably not identical. Nevertheless, future studies are necessary to further clarify the extent to which SON negativity can be considered a response separate from other ERP components.

## Conclusion

Given the informational value of the SON stimulus in our daily life, it is not surprising that the adult human brain has acquired a special capacity for detecting it fast and automatically. While personally meaningful names are distinguished from other unmeaningful names by slow neural processes as indexed by LN (>300 ms), SON is distinguished not only from unfamiliar names but also from familiar names in the early (<300 ms) preattentive stage of auditory processing [8]. The present findings are consistent with the notion that SON negativity represents a specific ERP measure of the early preattentive detection of SON. Further empirical and theoretical investigations are necessary to confirm (1) the validity of interpreting SON negativity as a distinct ERP component that is separate from other well studied waves, and (2) the degree to which this response is specific to SON.

## References

[1] Pavlov IP (1927). Conditioned reflexes.

[2] Sokolov EN (1963). Higher nervous functions: the orienting reflex. Ann Rev Physiol.

[3] Berlad I, Pratt H (1995). P300 in response to the subject’s own name. ElectroencephalogrClin Neurophysiol.

[4] Müller HM, Kutas M (1996). What’s in a name? Electrophysiological differences between spoken nouns, proper names and one’s own name. Neuroreport.

[5] Folmer RL, Yingling CD (1997). Auditory P3 responses to name stimuli. Brain Lang.

[6] Perrin F, Maquet P, Peigneux P, Ruby P, Degueldre C, Balteau E (2005). Neural mechanisms involved in the detection of our first name: a combined ERPs and PET study. Neuropsychologia.

[7] Holeckova I, Fischer C, Morlet D, Delpuech C, Costes N, Mauquiere F (2008). Subject’s own name as a novel in a MMN design: A combined ERP and PET study. Brain Res.

[8] Tateuchi T, Itoh K, Nakada T (2012). Neural mechanisms underlying the orienting response to subject’s own name: An event-related potential study. Psychophysiology.

[9] Pratt H, Berlad I, Lavie P (1999). ‘Oddball’ event-related potentials and information processing during REM and non-REM sleep. Clin Neurophysiol.

[10] Perrin F, García-Larrea L, Mauguiere F, Bastuji H (1999). A differential brain response to the subject’s own name persists during sleep. Clin Neurophysiol.

[11] Perrin F, Goldman S, Moonen G (2006). Brain response to one’s own name in vegetative state, minimally conscious state, and locked-in syndrome. Arch Neurol.

[12] Qin P, Di H, Yan X, Yu S, Yu D, Laureys S (2008). Mismatch negativity to the patient’s own name in chronic disorders of consciousness. Neurosci Lett.

[13] Fischer C, Dailler F, Morlet D (2008). Novelty P3 elicited by the subject’s own name in comatose patients. Clin Neurophysiol.

[14] Fischer C, Luaute J, Morlet D (2010). Event-related potentials (MMN and novelty P3) in permanent vegetative or minimally conscious states. Clin Neurophysiol.

[15] Schnakers C, Perrin F, Schabus M, Majerus S, Ledoux D, Damas P (2008). Voluntary brain processing in disorders of consciousness. Neurology.

[16] Schnakers C, Giacino JT, Løvstad M, Habbal D, Boly M, Di H (2014). Preserved covert cognition in noncommunicative patients with severe brain injury?. Neurorehabil Neural Repair.

[17] Näätänen R, Paavilainen P, Rinne T, Alho K (2007). The mismatch negativity (MMN) in basic research of central auditory processing: a review. Clin Neurophysiol.

[18] Oldfield RC (1971). The assessment and analysis of handedness: the Edinburgh inventory. Neuropsychologia.

[19] Arao H, Suwazono S (2014). ERP responses to unattended own names: effects of emotion and experimental paradigms. Int J Psychophysiol.

[20] Girodo M, Deck TP, Campbell KB (2002). Event-related potentials reveal the effects of altering personal identity. Neuroreport.

[21] Zhao K, Yuan J, Zhong Y, Peng Y, Chen J, Zhou L (2009). Event-related potential correlates of the collective self-relevant effect. Neurosci Lett.

[22] Paulmann S, Kotz SA (2008). Early emotional prosody perception based on different speaker voices. Neuroreport.

[23] Anderer P, Semlitsch HV, Saletu B (1996). Multichannel auditory event-related brain potentials: effects of normal aging on the scalp distribution of N1, P2, N2 and P300 latencies and amplitudes. Electroencephalogr Clin Neurophysiol.

[24] Wunderlich JL, Cone-Wesson BK, Shepherd R (2006). Maturation of the cortical auditory evoked potential in infants and young children. Hear Res.

[25] Itoh K, Suwazono S, Nakada T (2010). Central auditory processing of noncontextual consonance in music: an evoked potential study. J Acoust Soc Am.

[26] Itoh K, Nakada T (2013). . Human brain detects short-time nonlinear predictability in the temporal fine structure of deterministic chaotic sounds. Phys Rev E Stat Nonlin Soft Matter Phys.

